# Multi-epitope chimeric vaccine designing and novel drug targets prioritization against multi-drug resistant *Staphylococcus pseudintermedius*

**DOI:** 10.3389/fmicb.2022.971263

**Published:** 2022-08-04

**Authors:** Samavia Jaan, Mohibullah Shah, Najeeb Ullah, Adnan Amjad, Muhammad Sameem Javed, Umar Nishan, Ghazala Mustafa, Haq Nawaz, Sarfraz Ahmed, Suvash Chandra Ojha

**Affiliations:** ^1^Department of Biochemistry, Bahauddin Zakariya University, Multan, Pakistan; ^2^Institute of Food Science and Nutrition, Bahauddin Zakariya University, Multan, Pakistan; ^3^Department of Chemistry, Kohat University of Science and Technology, Kohat, Pakistan; ^4^Department of Plant Sciences, Faculty of Biological Sciences, Quaid-i-Azam University, Islamabad, Pakistan; ^5^Department of Basic Sciences, University of Veterinary and Animal Sciences Lahore, Narowal, Pakistan; ^6^Department of Infectious Diseases, The Affiliated Hospital of Southwest Medical University, Luzhou, China

**Keywords:** subtractive proteomics, reverse vaccinology, multi-drug resistance, *Staphylococcus pseudintermedius*, biofilm

## Abstract

Biofilm synthesizing multi-drug resistant *Staphylococcus pseudintermedius* bacteria has been recognized as the human infectious agent. It has been detected in the diseases of skin, ear, and postoperative infections. Its infections are becoming a major health problem due to its multi-drug resistance capabilities. However, no commercial vaccine for the treatment of its infections is currently available in the market. Here we employed the subtractive proteomics and reverse vaccinology approach to determine the potential novel drug and vaccine targets against *S. pseudintermedius* infections in humans. After screening the core-proteome of the 39 complete genomes of *S. pseudintermedius*, 2 metabolic pathways dependent and 34 independent proteins were determined as novel potential drug targets. Two proteins were found and used as potential candidates for designing the chimeric vaccine constructs. Depending on the properties such as antigenicity, toxicity and solubility, multi-epitope based vaccines constructs were designed. For immunogenicity enhancement, different specific sequences like linkers, PADRE sequences and molecular adjuvants were added. Molecular docking and molecular dynamic simulation analyses were performed to evaluate the prioritized vaccine construct’s interactions with human immune cells HLA and TLR4. Finally, the cloning and expression ability of the vaccine construct was determined in the bacterial cloning system and human body immune response was predicted through immune simulation analysis. In conclusion, this study proposed the potential drug and vaccine targets and also designed a chimera vaccine to be tested and validated against infectious *S. pseudintermedius* species.

## Introduction

Staphylococcus Intermedius Group (SIG) comprises four species, including *S. intermedius*, *S. delphini*, *S. cornubiensis*, and *S. pseudintermedius*. Among them, *S. pseudintermedius* is the predominant member of this group that causes infections in humans and other animals including dogs etc. It is an invasive zoonotic bacterium that has been linked with diseases of the epidermis, auditory and postoperative infections in humans (Garbacz et al., 2013). The documented infections are those involving endocarditis, surgical wounds, and catheter-related bacteremia. It is also known to cause secondary infections in the host with dermatitis resulting in skin modifications (Pompilio et al., 2015).

The first reported case of *S. pseudintermedius* contact with humans was described in 2006 as an endocarditis occurrence following implantation of an implantable cardioverter-defibrillator (ICD) in the patient (Van Hoovels et al., 2006). The most recent case of *S. pseudintermedius* has been reported in a 65-year-old male who underwent an allogeneic bone marrow transplant for acute lymphoblastic leukemia (Savini et al., 2013a). Although there are several antibiotics available for treatment of infection in dogs, the MRSP (Methicillin-Resistant *S. pseudintermedius*) is emerging with multi-drug resistance leading to severe infections and thus it is becoming difficult to avoid zoonotic pathogen transmission in humans. This indicates an urgent need of alternative therapies for near future threats (Savini et al., 2013b).

The biofilm formation is highly significant factor in all gram-positive Staphylococci infections as biofilm helps the pathogens to adhere at tissue surface or any other non-living materials. Moreover, the bacterial biofilms hold clinical importance because of their ability to confer antibiotic and disinfectant resistance, as well as resistance to host immune system in general, all of which promote infections in humans. All members of Staphylococcus Intermedius Group (SIG) are highly capable of biofilm formation that plays significant role in their pathogenicity and resistant phenomenon. Among them, the *S. pseudintermedius* strains can synthesize large quantities of biofilms that increase the drug resistance ability for their survival (Naserpour Farivar et al., 2014; Pompilio et al., 2015). The biofilm formation and antibiotic resistance phenomenon of *S. pseudintermedius* were discovered to be highly similar as of *S. aureus* indicating the possibility of same treatments to be effective. Thus, different studies have been investigated for possible drugs and vaccines identification through literature. The *S. aureus* and some other group members were found resistant to methicillin, amoxicillin, penicillin, and clavulanic acid drugs involving *macA*, *cna*, *fnbA*, and *fnbB* genes in experiments (Shahmoradi et al., 2019). Further studies also highlighted the wide range resistance phenomenon that emphasized the need for development of some novel drugs for SIG group infections. In other studies, different vaccines were also evaluated to inhibit biofilm activity of *S. aureus* and *S. epidermidis* where they discovered the PIA (Polysaccharide Intercellular Adhesion Antigen) as main biofilm producing substance (Mirzaei et al., 2016). The vaccines containing PIA were designed and subjected to testing, e.g., glycerol teichoic acid (Gly-TA) and polysaccharide intercellular adhesion (PIA) vaccine (Gholami et al., 2019), and PIA-rSesC conjugate vaccine for *S. aureus and S. epidermidis* (Mirzaei et al., 2019). The different strains of S. pseudintermedius have also been studied for examination of their biofilm producing abilities. Only few classes were discovered as weak whereas majority of the strains were highly capable of biofilm formation over which the studies of vaccine are needed to inhibit the *S. pseudintermedius* transmission (Mousavi et al., 2017; Bhooshan et al., 2020).

The *S. pseudintermedius* strains also contain invasion and virulence factors similar to *S. aureus* (Garbacz et al., 2013). The severe infection is assumed to be the combined effect of two essential virulence factors, including leucocidin (Luk-I) and Phenol soluble modulins (PSMs) (Maali et al., 2018). Luk-I consists of LukF-I and LukS-I and is linked with leukotoxicity in polymorphonuclear cells. It has a cytotoxic activity for receptor CXCR2. PSMs include δ-toxins and PSM as major virulent determinants. PSMs are hemolysins that disrupt the cell membrane of any cell type. Veterinary strains are capable of producing other virulence factors, including clumping factors, coagulases and DNases, protein A, lipase and β-hemolysin (Tanabe et al., 2013). β-hemolysin is neutral sphingomyelinase C involved in the hydrolysis of sphingomyelin in the membrane and thus causes damage to cells and tissues. Another virulence factor namely exfoliative toxin has been identified in canine pyoderma because its gene is mainly found in *S. pseudintermedius* isolated from infections. The *agr* quorum-sensing and signal transduction system plays a crucial role in the virulence regulation during infections (Van Duijkeren et al., 2011). Agr III is predominant in *S. pseudintermedius* species and is involved in the down regulation of surface-exposed proteins and up-regulation of proteins secreted. The *S. pseudintermedius* shows methicillin-resistance due to *spsO* gene expression in canine systems and is estimated to become more resistant in the future due to its similarity in the genome with MRSA (Methicillin Resistance Staphylococcus Aureus) that is already an emerging superbug (Pilla et al., 2013).

Less literature is available for *S. pseudintermedius* infections in humans followed by less knowledge about medications for its emerging drug resistance. It can be transmitted *via* pet animals that could become a future threat. It is predicted to become a superbug in future indicating its importance for designing alternative drug targets as well as potential vaccine candidates. In the current study we have employed different computational tools for identification of potential alternative druggable proteins. Furthermore, the so called immunoinformatics and reverse vaccinology approaches were used to design chimeric vaccines using the core-genome retrieved from publically available complete genomes of this species.

## Materials and methods

**FIGURE 1:**
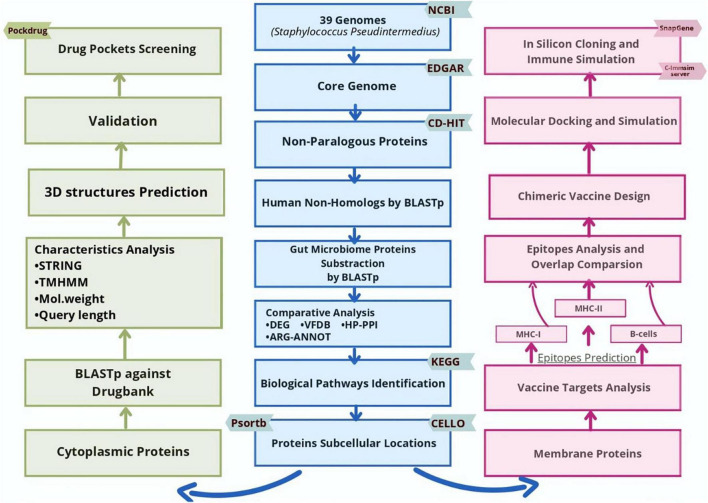
Flowchart of subtractive proteomics and reverse vaccinology approach followed for the potential drug and vaccine identifications against *S. pseudintermedius*.

This study was accomplished using several bioinformatics tools to address and prioritize substantial therapeutic drug and vaccine candidates for *S. pseudintermedius* species using core-proteome (Figure 1).

### Retrieval of core proteome dataset

Thirty-nine complete proteomes of *S. pseudintermedius* strains were retrieved from the Genbank server (Supplementary Table Ia). The core proteome of the retrieved datasets was prepared using EDGAR server (Gerdes, 2003). For this purpose, the thirty-nine complete proteomes were imported from GenBank to the EDGAR server and the representative genome, i.e., strain SP_11304-3A was selected as a reference. The Core-proteome contained all the shared proteins having essential activities in all the strains and consequently contributing to the survival of the species. The FASTA sequences of the core proteins were retrieved from GenBank and subjected to further analysis.

### Identification of non-paralogous proteins

Paralogous proteins are related *via* identical regions and have similar functions; thus, the core proteome screening was initiated by removing these proteins from the data to eliminate the redundancy using a CD-HIT online server (Huang et al., 2010). The band of 20 amino acids was chosen while other parameters like alignment coverage were kept at default. A threshold of 0.6 (i.e., 60%) was used for sequences identity, and paralogous proteins obtained by CD-HIT were evaded as per our previous study (Shah et al., 2020).

### Determination of human non-homologous proteins

The analysis was further proceeded by the determination of the identical proteins with the host proteome. This comparative analysis was performed using standalone BLASTp tool against the non-redundant *H. sapiens* database retrieved from the Uniprot server. As performed in our recent studies (Tahir Ul Qamar et al., 2020), the cutoff values of bit-score ≥100, percentage identity ≥35%, query coverage ≥35%, e-value < 1e-20 were selected as the baseline, and homologous proteins were filtered manually using the venny server (Aslam et al., 2020). Furthermore, the resulted non-homologous proteins were also subtracted from the human gut microbiome with the BLASTp criterion as identity ≥50 and e- > 1e-04. The homologs were removed and final non-homologous proteins were selected for further assessment to seek out potential drug targets.

### Retrieval of essential, virulent, drug-resistant, and host-interacting proteins

Essential genes are effective drug targets because of their importance in survival mechanisms. Their analysis helps identify the minimal set of self-sufficient proteins conserved in pathogen and necessary for existence. Essential protein screening was done against the Database of Essential Genes (Zhang et al., 2004). The analysis was performed with cutoff values: bit-score ≥100, e-value of 1e-4, and ≥35% identity. Likewise, virulent factor proteins have a significant role in pathogens infecting the host organism, and thus determination of these factors is also an important criterion for drug discovery. For the determination of the virulent factors, the proteins were scanned using standalone BLASTp against the VFDB (Liu et al., 2019).

Bacterial species are gaining resistance to available antibiotics, and the ARG-ANNOT repository has cataloged these resistant drug targets (Gupta et al., 2013b). Hence, BLASTp was used to locate antibiotic resistance targets from the ARG-ANNOT database. The host-pathogen relationship is also necessary for the infectious pathogen’s survival inside the host system thus this screening was performed using HP-PPI database (Barh et al., 2013). Finally, the prioritized protein targets were obtained by combining the findings from all these analyses.

### Metabolic pathways analysis of prioritized proteins

Human host non-homologous and pathogen specific protein targets were further checked using the KEGG database for the involvement in distinctive metabolic pathways of *S. pseudintermedius*. The KEGG identifiers were obtained *via* the KAAS server, and a KEGG pathway search was performed to find the pathways linked with the pathogen. The *S. pseudintermedius* pathways were manually compared with the metabolic pathways of *H. sapiens* to find common and unique pathways. The common pathway proteins were omitted to evade interference with host pathways.

Furthermore, the unique pathways were further evaluated to discover proteins engaged in common pathways and then subtracted to identify actual unique proteins. These pathogen-specific proteins were termed KEGG-dependent proteins because they were believed to be involved in only pathogen survival pathways. The remaining KEGG pathway-independent proteins not associated in either route were also treated separately since they might be necessary for processes unrelated to metabolic pathways. These identified proteins were further analyzed to predict potential therapeutic drug targets.

### Determination of sub-cellular localization

PSORTb v3.0 server was used to predict the subcellular location of the resultant key target proteins. Although the PSORTb results comprised cytoplasmic and membrane protein localization prediction, but its results are restricted to only some localization sites. Hence, CELLO v2.5 was further used to validate sub-cellular locations. Sub-CELlular Localization predictor (CELLO) envisages the location with functional gene ontology annotation and helps determine the biological significance of proteins (Tahir Ul Qamar et al., 2020). Cytoplasmic proteins were chosen as drug target candidates, whereas membrane and extracellular proteins were employed to evaluate subunit vaccine targets (Solanki and Tiwari, 2018).

### Identification of final potential drug targets

Druggability analysis of the shortlisted proteins was performed using the Drugbank database (Wishart et al., 2006). The proteins with a bit score ≥100 and an *E*-value < 0.005 were designated as FDA-approved drug targets. In contrast, the remaining proteins were chosen as novel drug targets and subjected to molecular weight prediction (Protparam tool), STRING analysis and TMHMM helix prediction. Low-molecular-weight protein targets (less than 100–110 kDa) are preferred over high-molecular-weight proteins because they have better accessibility to solvent (Wishart et al., 2006). The Protein-protein interactions were analyzed by using STRING database (Mering et al., 2003), where the hub proteins were determined by selecting the average node degree greater than 5.0 (*K* ≥ 5) (Aslam et al., 2020). TMHMM, which identifies trans-membrane helices in proteins, was used to rule out proteins with multiple helices. The membrane-spanning helices were limited to 0 or 1 since protein with more than one helix is challenging to clone in the host system and purify for use in the laboratory (Hosen et al., 2014). Proteins with >800 amino acids are difficult to manipulate in the lab and hence proteins with query lengths of less than 800 amino acids were shortlisted.

### Prediction of 3D structures and drug pockets

The BLASTp server was used for the search of 3D homologs list of all shortlisted drug targets. It compared query protein sequences with the protein sequences of known folds in PDB (Protein Data Bank) database. The homologs with more than 60% similarity were preferred and ERRAT server was used for their validation. Finally, the PockDrug server (Hussein et al., 2015) was applied to analyze drug binding pockets in preferred 3D structures and their affinity with the drugs.

### Identification of potential vaccine targets

The shortlisted membrane and extracellular proteins were characterized using the ProtParam tool, accessible on the Expasy server. This tool identifies many physicochemical characteristics of input proteins, such as molecular weight, theoretical pI, aliphatic index, GRAVY, and the total number of amino acids. TMHMM server was also used to identify the transmembrane helices, with a prioritized value of 0 or 1. Vaxijen server v2.0 (Doytchinova and Flower, 2007) and AllergenFP (Dimitrov et al., 2014) were used to evaluate the vaccine candidates for antigenicity and allergenicity, respectively. In the case of Vaxijen, the cutoff value was selected as >0.5, and the accuracy rate was set at default (70–89%).

### T-cell epitopes prediction

T-cells are part of the adaptive immune response, and determining the T-cells binding epitopes is a critical step for vaccine development. These T-cell epitopes interact with the Antigen-Presenting Cells (APCs) attached with Major Histocompatibility Complexes (MHCs) (Sanchez-Trincado et al., 2017). Prediction of MHC binding epitopes is significant as they are vital carriers of antigenic determinants and aids in determining high affinity T-cell binding epitopes. The NetMHCpan-4.1 (IEDB-AR) (Reynisson et al., 2020) was employed for MHC-I binding predictions where the length of predicted peptides was kept as default, i.e., 9-mer, and the HLA alleles set was used, including >97% of population coverage. For MHC-II binding predictions, IEDB recommended 2.22 method was selected. The length of predicted epitopes was set as 15-mer, and a reference set of >99% population coverage was used for MHC-II predictions. The resulting T-cell epitopes were selected based on either IC50 values or percentile ranks. Lower values of IC50 or rank determine the higher affinity of epitopes with MHC molecules.

### Evaluation of predicted T-cell epitopes

To select the final epitopes for the vaccine construct, the promiscuous T-cell epitopes were scanned using various criteria. The Immune Epitope Database (IEDB) (Vita et al., 2019) was used to complete the conservancy study where epitopes having a conservation rate of more than 50% were considered. Vaxijen v2.0 (Doytchinova and Flower, 2007) and Toxinpred server (Gupta et al., 2013a) were used to analyze the antigenicity and toxicity of these epitopes. The epitopes with antigenicity values >0.4 and non-toxic features were selected. Using the IEDB Class I immunogenicity tool, an additional parameter of immunogenicity was evaluated for MHC-I predicted epitopes using default values. The epitopes that obtained positive values were selected. Interferon-gamma, Interleukin-4, and Interleukin-10 induction were also analyzed concerning MHC-II epitopes because of their association with eliciting B-cell responses (Kim et al., 2001). Those epitopes were selected that had at least one type of induction ability. After evaluating all the parameters, final epitopes were deemed as promiscuous T-cell epitopes. Moreover, additional analysis of resultant HLA alleles was also performed using the MHC cluster v2.0 server (Thomsen et al., 2013). This server helps to cluster the resulted MHC molecules functionally and identify their positions in commonly occurring HLA supertypes. The results are visualized in the form of heat maps.

### B-cell epitopes prediction

Antibodies generated by B-cells can access the solvent-exposed portions of pathogen proteins, resulting in an immunological response. As a result, B-cell epitopes are expected to be useful in vaccine development. Herein, several web servers including BCPred, AAP, FBCPred, and Bepipred, were used to predict linear B-cells epitopes. For antigenicity calculations of predicted B-cell epitopes, Vaxijen v2.0 was used with a threshold of >0.4. The antigenic B-cell epitopes were assessed for overlaps and then combined to make the final B-cell epitopes. Various biophysiochemical characteristics were identified, including flexibility prediction by Karplus and Schulz method, beta-turn prediction by Chou-Fasman method, linear epitopes prediction using BepiPred, antigenicity prediction by Kolaskar Tongaonkar method, hydrophilicity analysis by Parker method, and surface accessibility analysis of proteins by Emini calculations, as well as the IEDB’s ElliPro server for linear and conformational B-cell epitopes (Solanki and Tiwari, 2018).

### Multi-epitope chimeric vaccine construction

The overlapping B and T-cell epitopes were selected for the construction of the chimeric vaccines. For this purpose, the selected epitopes were combined in different combinations to design constructs. The first vaccine construct was made using epitope sequences 51–72, 124–148, 197–218, 265–288 of WP_014613729.1 and 4–34, 100–119, 143–162 of WP_130921585.1 protein with the help of amino acids linkers. Secondly, epitope sequences 76–121, 151–188, 227–251 of WP_014613729.1 and 58–98, 143–162, 202–239 of WP_130921585.1 were joined using linkers to make the second vaccine construct. To improve the vaccine efficacy, two vaccine constructs were combined with four distinct adjuvants namely ribosomal protein L7/L12 (WP 088359560.1), HBHA Adjuvant (AGV15514.1), HBHA conserved sequence, and Beta-defensin 3. The PADRE sequence was also introduced to boost vaccine construct effectiveness. Epitopes were joined together with GGGS and HEYGAEALERAG linkers, while adjuvant sequences were coupled with EAAAK linkers at N- or C-terminus of vaccine constructs. GGGS linkers were also used to attach PADRE sequences.

### Vaccine constructs analysis

The vaccine constructs were evaluated using numerous factors to determine the best candidates for final chimeric vaccine development. Expasy Protparam server was used to determine the vaccine constructs’ molecular weight, total amino acids in the query sequence, instability and aliphatic index, theoretical isoelectric point, and GRAVY score. Vaxijen v2.0 server with a threshold of >0.75 and ANTIGENpro server with a score greater than 0.90 were used to calculate antigenicity of vaccine constructs. The allergenicity was assessed using the AllergenFP webserver, which employs a four-step algorithm to predict allergen epitopes. The SOLpro server (Magnan et al., 2009) was selected to predict vaccine construct solubilities during heterologous expression inside an *E. coli* host. The acceptable solubility overexpression threshold was kept as >0.5 (Solanki and Tiwari, 2018). The PSIPRED server was used to generate the secondary structure using PSI-BLAST output files in two feed neural network systems (McGuffin et al., 2000). The RaptorX property server (Wang et al., 2016) was additionally used to estimate the percentages of secondary structure and solvent accessibility (ACC) of vaccine construct regions. By employing the devised techniques for computing, regions forming helix, beta-strand, and coil structures were predicted, plus solvent-exposed and buried regions were also examined. The tertiary structure was then predicted using the Phyre2 server (Kelley et al., 2015), which was subsequently refined by the Galaxyrefine server (Heo et al., 2013). Proteins with disulfide linkages have the better three-dimensional conformation and are more stable in nature thus disulfide bonds were introduced through Disulfide by Design 2.0 (Dbd2) server (Craig and Dombkowski, 2013).

### Molecular docking and dynamic simulation

The interactions of the final vaccine construct with the HLA allele receptors (MHC molecules in *H. sapiens*) were analyzed using the PatchDock server (Schneidman-Duhovny et al., 2005). The final vaccine construct was docked with four distinct frequently occurring HLA alleles: 1A6A (HLA-DR B1*03:01), 3C5J (HLA-DR B3*02:02), 1AQD (HLA-DRB1*01:01), and 5NI9 (HLA-DR B1*04:01). The docking results were optimized using the FireDock feature (Mashiach et al., 2008). By rearranging and re-scoring the docking complexes, the FireDock tool optimizes the results. It generates the top ten refined complexes using the global binding energy values that have been rechecked and modified. The vaccine construct (V2) was additionally docked with the TLR 4 (2Z63) complex using the PatchDock server. The FireDock server re-scored the top ten models of resulting complexes and the binding energy of the improved candidates was used to rank them. The best interacting complex with the highest docking score was further verified by dynamics simulation methodology that describes the behavior of molecules in a natural environment. Since the iMOD server (Lopéz-Blanco et al., 2011) is quick and effective, we used it to explore and evaluate the interaction between the proposed vaccine construct and its best-interacted receptor, i.e., TLR4 complex. This program uses four essential factors to predict the orientation and range of the protein-protein complex’s fundamental motions: deformability, eigenvalues, B-factors, and covariance.

### Codon adaptation and *in silico* cloning

The JCAT tool (Grote et al., 2005) was used for converting codons in the vaccine construct according to the expression system. The vaccine construct sequence was changed into a DNA sequence by back-translation, and the Codon Adaptation Index (CAI) values were used to guide the adaptation. Several cleavage positions of various restriction enzymes, bacterial ribosome binding sites, and rho-independent transcription terminators, were all deselected. To ensure vaccine construct expression, the SnapGene tool was employed to perform *in silico* cloning in the pET28a (+) vector of *E. coli* host (Aslam et al., 2020).

### Immune simulation

C-ImmSim server (Castiglione and Bernaschi, 2004) was used for the execution of computational immune simulations to better assess the vaccine construct effectiveness and immunological profile. Volume (50), random seed (12345), number of steps (1050), and the total number of injections (01, 84, and 170) were all considered settings for the immune simulations. The remaining options were kept as default.

## Results

### Core-proteome retrieval and non-homologous proteins determination

The proteomes of thirty-nine *S. pseudintermedius* strains were obtained from the NCBI database and analyzed through the EDGAR server for core proteome formation. The EDGAR database generated 1386 core proteins (Supplementary Table 1b), which were then employed for subtractive proteomics and reverse vaccinology approach. The CD-HIT clusters out paralogous proteins that complicate results due to their high sequence similarity and comparable activities. Consequently, only non-paralogous protein targets remain in the data, reducing the number of proteins and allowing the identification of limited but more selective therapeutic drug targets (Shah et al., 2020). As a result, three paralogous proteins were removed and subsequent proteins subjected to further analysis.

Host non-homologous proteins are crucial in determining therapeutic targets because of their uniqueness in a particular organism compared to the host. For this purpose, the non-paralogous proteins were subjected to BLASTp against the proteome dataset of *H. sapiens*, and a total of 59 homologous proteins were filtered out due to their similarity with the host. Subtraction of gut flora related proteins was also undertaken since the gut flora comprises many critical microorganisms engaged in digestion and other vital processes. The resulting 956 pathogen specific proteins were selected as lead drug candidates for further analysis.

### Essential, virulent, antibiotic-resistant, and host-interacting proteins

Essential genes are a minimal set of genes that play a decisive role in the viability and development of an organism. These are sustained in principal biological functions and pave the way for predicting the effective potential drug targets. DEG database contain record of the available essential elements, and thus we used it to predict the essential genes in our dataset. The BLASTp against DEG database filtered out 242 essential proteins that were significant for the survival of *S. pseudintermedius*. Due to the essentiality of these proteins for the bacterial survival, they are considered significant in drug development. Moreover, the study of virulence factor proteins revealed the importance of bacteria in numerous illnesses, necessitating the hunt for new virulence factors (VFs). The VFDB (Virulence Factor Database) findings indicated that 20 proteins were associated with *S. pseudintermedius* pathogenicity.

Similarly, ARG-ANNOT (Antibiotic Resistance Gene-ANNOTation) database is an updated informatics library containing about 1,689 known antibiotic resistance genes. It is utilized to look at existing and emerging drug resistance in genomic sequences (Zankari, 2014). This resulted in prediction of 8 proteins being involved in the breakdown of antibiotics and their efflux. Another essential component considered for a drug target to be effective is the interaction between the pathogen and the host. Some proteins are crucial in pathogen survival in the host system, and their identification will aid in the development of more specific therapeutic targets against *S. pseudintermedius* species. These proteins are determined through the host-pathogen protein-protein interactions (HP-PPI) database. The search in this database revealed 2 proteins to be engaged in the host’s interactive pathways. After integrating all these analysis, a total of 255 potential drug targets were discovered. These proteins may be the critical targets for limiting infection of *S. pseudintermedius* species.

### Metabolic pathways analysis and localization of potential drug targets

The resulting proteins were analyzed using the KEGG database to determine the alternative and common pathways. A total of 102 pathways were retrieved from KEGG, out of which 26 bacterial unique pathways were identified that revealed the involvement of 58 unique and 99 common proteins. Following the analysis, 7 distinct proteins were identified as KEGG-dependent potential therapeutic targets (Table 1). To determine KEGG pathways independent proteins, both common and unique pathway proteins were integrated and subtracted from the proteins obtained during the previous step. A total of 149 proteins were found as metabolic pathways independent proteins. The resulting KEGG-dependent and independent proteins could be potential therapeutic targets against *S. pseudintermedius*. Subcellular location is an important attribute in determining the biological functions of different proteins. The accessibility of these proteins to drugs is highly dependent on their location. Subcellular localization results revealed 6 KEGG-dependent and 136 KEGG-independent cytoplasmic proteins. These were considered drug candidates due to their solvent accessible properties and ease of extraction, isolation, and development in the laboratory (Solanki and Tiwari, 2018). A total of 6 membrane proteins that were part of the cell wall were predicted, while 8 extracellular proteins were identified. The membrane and extracellular proteins were processed as vaccine candidates for chimeric vaccine production as the surface-exposed proteins and secreted proteins are considered excellent vaccine targets as they can be rapidly recognized by the immune cells (Knox et al., 2003).

**TABLE 1:** *S. pseudintermedius* proteins involved in the unique metabolic pathways.

Sr No.	Protein IDs	Protein names	KO identifiers	Unique pathway names	Pathway IDs
1	WP_096536529.1	S1C family serine protease	K04771	Cationic antimicrobial peptide (CAMP) resistance	SSD01503
2	WP_014613729.1	N-acetylmuramyl-L-alanine amidase	K01448	Cationic antimicrobial peptide (CAMP) resistance	SSD01503
3	WP_015728718.1	undecaprenyl-diphosphate phosphatase	K06153	Peptidoglycan biosynthesis	SSD00550
4	WP_014614850.1	response regulator transcription factor	K07667	Quorum sensing	SSD02024
5	WP_096533404.1	ABC transporter permease	K02034	Quorum sensing	SSD02024
6	WP_014614877.1	ABC transporter ATP-binding protein	K02031	Quorum sensing	SSD02024
7	WP_015729294.1	HAMP domain-containing sensor histidine kinase	K07636	Two-component system	SSD02020

### Final potential drug targets against *S. pseudintermedius*

**FIGURE 2:**
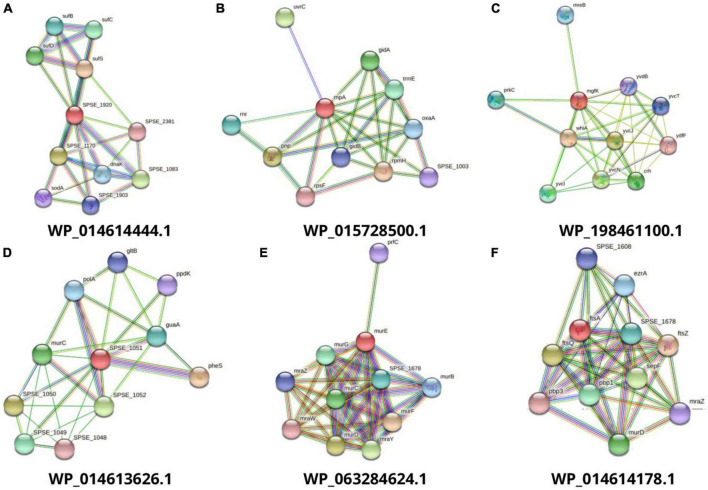
Interaction analysis of predicted drug targets with other proteins using STRING database where query proteins are indicated by red color. The proteins with the best predicted three-dimensional structures **(A to F)** are shown to summarize the drug target’s PPI list.

After the druggability analysis, 119 KEGG-independent proteins were matched with FDA-approved druggable targets, while the others were assessed as novel drug candidates. Further analysis for validation of these novel drug targets was performed by determining their molecular weights, lengths, protein-protein interactions, and membrane spanning helices. The proteins with molecular weight <110 were assumed to be the best candidates for drug targets because of solvent accessibility and purification facility in practical implementation (Soltan et al., 2021). The STRING database helps to study the functional interactions of protein expression. Out of these drug targets, proteins with an average node degree value (K) greater than five were considered as hub proteins (Figure 2). For the convenience of drug target production in the lab, query length with less than 800 amino acids and helices composition of 0 or 1 are preferred (Aslam et al., 2020). A total of 36 potential final drug targets were obtained, including 2-KEGG-dependent and 34 KEGG-independent proteins after prioritization and filtration through the stated tools (Table 2).

**TABLE 2:** Metabolic pathways dependent and independent potential drug target proteins of *S. pseudintermedius.*

Sr No.	Protein ID	STRING (K > 5)	TMHMM	Molecular weight (Da)	Query Length
**Pathways Dependent Proteins**					
1	WP_014614877.1	7.27	0	30641.49	272
2	WP_096536529.1	5.82	1	45127.68	423
**Pathways Independent Proteins**					
1	WP_014613335.1	5.09	0	38950.32	350
2	WP_014614444.1	5.09	0	16703.98	149
3	WP_015728500.1	5.09	0	13481.01	115
4	WP_198461100.1	5.09	0	36453.7	331
5	WP_014613337.1	5.45	0	19202.97	175
6	WP_014613626.1	5.45	0	21907.04	199
7	WP_014613059.1	5.64	0	24290.12	216
8	WP_019165903.1	5.64	0	16761.96	146
9	WP_014614642.1	5.82	0	29321.13	258
10	WP_014614184.1	6	1	14887.24	128
11	WP_014613696.1	6.36	0	23211.99	203
12	WP_070407479.1	6.55	0	31551.41	273
13	WP_014613689.1	6.91	0	35772.07	307
14	WP_014613910.1	6.91	0	12851.48	111
15	WP_037542841.1	6.91	0	38036.77	341
16	WP_014614215.1	7.09	0	20501.87	179
17	WP_014614952.1	7.09	0	32613.38	283
18	WP_015728743.1	7.64	0	71964.63	625
19	WP_099991805.1	7.64	0	49172.07	413
20	WP_014613542.1	7.82	0	17730.26	154
21	WP_063284624.1	8	0	54065.44	494
22	WP_014613764.1	8.36	0	22437.58	194
23	WP_014613960.1	8.36	0	49361.54	419
24	WP_014612662.1	8.55	0	41956.74	377
25	WP_020219473.1	8.55	0	21958.1	194
26	WP_014614178.1	8.73	0	52106.02	466
27	WP_014614625.1	8.91	0	29789.3	264
28	WP_037543791.1	8.91	0	43597.1	382
29	WP_100006538.1	9.27	0	24690.04	213
30	WP_015728743.1	9.64	0	71964.63	625
31	WP_014613585.1	9.82	1	35185.22	303
32	WP_014613759.1	9.82	0	20402.62	175
33	WP_020219604.1	10	0	19910.5	171
34	WP_081248043.1	10	1	56927.83	509

### Structure predictions and drug pockets screening of potential drug targets

For the determination of the 3D structures, the target proteins were searched in PDB database. Six proteins showed >60% similarity with the proteins of the reported 3D structures. These proteins were modeled using the SWISS-MODEL online server, and the ERRAT was employed to validate these predicted structures. They were analyzed for drug pockets using the PockDrug server. The PockDrug server uses a variety of the pocket estimating approaches to predict druggable pocket based on the structure of the protein and ligands contact estimation. The threshold of >0.5 was kept regarding residues in the pocket for site selection as drug pocket in drug target proteins (Table 3).

**TABLE 3:** Druggability screening of the prioritized potential drug targets.

Protein IDs	Protein names	PDB homologs	% Identity	Query coverage (%)	Q-mean scores	GMQE score	Template vs. Model RMSD (Å)	ERRAT scores	Pocket residues (PockDrug > 0.5)
WP_014614444.1	SUF system NifU family Fe-S cluster assembly protein	6jzv.1.A	67.8	98	0.77	0.78	2.00 vs. 2.00	96.8992	16.0 (1.0)
WP_015728500.1	MULTISPECIES: ribonuclease P protein component	6ov1.2.A	78.07	99	0.83	0.86	1.70 vs. 1.66	95.2381	17.0 (0.94)
WP_198461100.1	YvcK family protein	2ppv.1.A	67.48	99	0.86	0.9	2.00 vs. 2.00	98.4177	19.0 (0.81)
WP_014613626.1	DUF4479 domain-containing protein	3bu2.1.A	64.65	99	0.82	0.88	2.70 vs. 2.70	92.0245	20.0 (0.72)
WP_063284624.1	UDP-N-acetylmuramoyl-L-alanyl-D-glutamate–L-lysine ligase	4c12.1.A	79.47	100	0.91	0.94	1.80 vs. 1.80	94.7034	30.0 (0.71)
WP_014614178.1	Cell division protein FtsA	3wqt.1.A	75.32	99	0.88	0.83	2.20 vs. 2.20	92.3944	No Pockets

### Selection of vaccine target candidates

The proteins with a molecular weight of 30–110 kDa, theoretical pI > 7.0, aliphatic index > 70, GRAVY negative factors, and amino acid length of less than 800 were selected as the top vaccine candidates. Vaccine target proteins were further analyzed using the Vaxijen server for antigenic protein prediction and AllergenFP for allergic proteins identification. The target proteins having antigen probability score more than 0.5 and showing non-allergic behavior could compete as suitable vaccine targets (Barh and Kumar, 2009). After screening, two extracellular proteins (WP 014613729.1 and WP 130921585.1) were identified as best potential vaccine target candidates.

### Selection of T-cell epitopes for chimeric vaccine

The anticipated vaccine must evoke an immunological response in the host organism; hence T-cell and B-cell epitopes identification is required for vaccine designing. T-cells are classified as either CD4 + (HTLs) or CD8 + (CTLs) depending on the special receptors on the membranes. The T-cells binds to epitopes are presented by two variants of Major Histocompatibility Complexes (MHC), i.e., MHC-I and II molecules. CTLs can bind to MHC-I binding epitopes; however, HTLs interact with MHC-II molecules. When antigenic epitopes interact more strongly with MHC molecules, the immune response is more robust, hence predicting top affinity epitopes is significant (Sanchez-Trincado et al., 2017). Both MHC molecule binding epitopes for vaccine targets were determined utilizing the IEDB database. Accordingly, NetMHCpan-4.1 was used to determine MHC-I interacting epitopes, and peptides having a rank of less than 0.2 were shortlisted. From the proteins WP 014613729.1 and WP 130921585.1, a total of 44 and 46 MHC-I binding epitopes were identified, which were then examined for overlaps. Top 10 non-overlapping MHC-I binding epitopes of both proteins were proceeded for further analysis (Supplementary Table 2). In the case of MHC-II binding prediction, the IEDB-recommended 2.22 approach was utilized, and epitopes with a rank of less than 10.0 were selected. As a result, 166 and 152 MHC-II binding epitopes were determined for WP 014613729.1 and WP 130921585.1, respectively. The top 10 non-overlapping MHC-II binding epitopes were designated for further analysis (Supplementary Table 2). To confirm the presence of resulting MHC alleles in majority of population, MHCluster v2.0 server was employed (Solanki and Tiwari, 2018). This server assessed the resulting interacting alleles clusters that were depicted in the heat map presentation (Supplementary Figure 1).

### Analysis of predicted T-cell epitopes

Although the predicted T-cell epitopes have a strong affinity with MHC-I and MHC-II molecules, this may not indicate if the epitopes can trigger an immune reaction; hence various parameters were used to further analyze the epitopes. Conservancy analysis was used to find conserved epitope sequences across species genotypes, allowing the vaccine to be employed against a variety of strains. More than 50% conservation value was selected for effective epitopes on various strains. Antigenicity (as determined by Vaxijen v2.0) and toxicity (as assessed by Toxinpred) were also calculated, with a threshold of >0.4 for Vaxijen and non-toxic characteristics for Toxinpred. In addition, the IEDB epitope immunogenicity tool for MHC-I immunogenic capability and different cytokines induction tools for MHC-II epitopes were used in the analysis (Solanki and Tiwari, 2018). As a result, 4 MHC-I and 7 MHC-II predicted epitopes were obtained for WP 014613729.1, while 2 MHC-I and 8 MHC-II predicted epitopes were generated for the WP 130921585.1 protein (Supplementary Table 3).

### Selection of B-cell epitopes for chimeric vaccine

The two vaccine candidate proteins were further utilized to predict different types of linear and conformational B-cells epitopes. Linear epitopes are easier to predict because they are estimated using primary sequences, but conformational epitopes require three-dimensional structures of proteins, which makes them difficult to identify owing to the unavailability of 3D structures for many proteins. The ability to predict both types of B-cells aids in the development of effective vaccines; thus, they were predicted using various servers for accuracy, including BCPred, AAP (Amino Acid Pair), FBCPred, and Bepipred. BCPred is built on 5 distinct kernel approaches cross-validated five times using Support Vector Machine (SVM) (EL-Manzalawy et al., 2008). BCPred predicted a total of 8 and 7 B-cell epitopes for WP 014613729.1 and WP 130921585.1, respectively. Of these, Vaxijen estimated 6 epitopes of WP 014613729.1 and 4 of WP 130921585.1 as antigenic peptides. FBCPred server (El-Manzalawy et al., 2008) uses the subsequent kernel method to predict flexible linear B-cell epitopes. This server predicted 6 and 8 B-cell epitopes for WP 014613729.1 and WP 130921585.1, respectively. As a result, 5 epitopes were predicted as antigenic peptides for WP 014613729.1 and WP 130921585.1, respectively. The AAP server is based on the likelihood that certain amino acid pairs repeat more frequently in the B-cell epitopes. As a result, this server predicts potent B-cell epitopes by combining the probability score with Support Vector Machine (EL-Manzalawy et al., 2008). AAP server predicted 7 B-cell epitopes for WP 014613729.1 and 6 for WP 130921585.1. Of which, Vaxijen identified 5 epitopes of WP 014613729.1 and all epitopes of WP 130921585.1 as antigenic in nature. The linear epitopes were also estimated combining biochemical parameters such as surface accessibility, amino acid composition, hydrophilicity, and hydrophobicity by accessing various tools on IEDB server. This server includes the various prediction methods, i.e., Karplus and Schulz Flexibility Prediction, Chou-Fasman beta-turn prediction, BepiPred linear epitope prediction, Kolaskar Tongaonkar antigenicity, Parker hydrophilicity prediction, and Emini surface accessibility prediction (Supplementary Figure 2). The ElliPro and Discotope server of the IEDB were used to identify B-cell conformational epitopes. Due to the lack of three-dimensional structure and homologs for the two vaccine candidates, conformational B-cells could not be developed. All projected B-cell epitopes were examined for overlaps in the last step where 7 and 6 final B-cell epitopes were obtained for WP 014613729.1 and WP 130921585.1, respectively (Supplementary Table 4).

By using final hypothesized B-cells epitopes as a baseline, T-cell epitopes of both proteins were evaluated for overlaps, and final epitopes for chimeric vaccine were shortlisted. As a result, all predicted B-cell epitopes (7 for WP 014613729.1 and 6 for WP 130921585.1) were determined to be overlapping and further proceeded to model the chimera vaccine (Supplementary Table 5).

### Chimeric vaccine constructs modeling

The final shortlisted epitopes were combined to form eight vaccine constructs. These vaccine constructs were termed as V1 to V8 after being attached with adjuvant sequences including L7/L12 (WP 088359560.1), HBHA Adjuvan t (AGV15514.1), HBHA conserved, and Beta-defensin 3, respectively. PADRE sequences were also incorporated in the vaccine constructs as these sequences activate CD4+ helper T-cells, which improve the peptide vaccine’s effectiveness (Ghaffari-Nazari et al., 2015). To promote antigen presentation activity, linkers are commonly used in the production of multi-epitope vaccine constructs. The KK, GPGPG, GGGS, EAAAK, and HEYGAEALERAG are common linkers employed in vaccine constructions (Negahdaripour et al., 2018). Herein, all eight vaccine constructs were linked using EAAAK, GGGS, and HEYGAEALERAG linkers (Table 4).

**TABLE 4:** Designed vaccine constructs with different adjuvant sequences.

S/No.	Vaccine constructs	Epitope sequences with adjuvants	Complete vaccine constructs sequences
1	V1	WP_014613729.1 (51–72, 124–148, 197–218, 265–288), and WP_130921585.1 (4–34, 100–119, 143–162) epitopes with L7/L12 Ribosomal protein adjuvant and PADRE sequence	EAAAKMSDLKNLAETLVNLTVKDVNELAAILKDEYGIEPAAAAVVMAGPGAEAAEEKTEFDVILK SAGASKLAVVKLVKDLTGAGLKEAKDMVDGAPAAIKSGISKDEAEALKKQLEEAGAEVELKEAAA KAKFVAAWTLKAAAGGGSDAEIRTGPNAAYPVLYQVHKGDGGGSLDPGHGGSDQGASSRQGDKT LEKDIGGGSLETPNANGATVYWFHEQQEGLAGGGSTVMMKDKNHRQIVEQAIVDGLESYGGGSA KFVAAWTLKAAAGGGSEIPKINNEYLKEKRKKQRIQQRRVQRMIVGIHEYGAEALERAGDEVTIEK GLFNPIEVNVKEHHEYGAEALERAGVGATFIPYDNVNNGQTSSASAKEVQSGTASEDKAKDDL QKALNKIKDEEHEYGAEALERAGAKFVAAWTLKAAAGGGS
2	V2	WP_014613729.1 (51–72, 124–148, 197–218, 265–288), and WP_130921585.1 (4–34, 100–119, 143–162) epitopes with HBHA Adjuvant and PADRE sequence	EAAAKMAENPNIDDLPAPLLAALGAADLALATVNDLIANLRERAEETRAETRTRVEERRARLTKF QEDLPEQFIELRDKFTTEELRKAAEGYLEAATNRYNELVERGEAALQRLRSQTAFEDASARAEGYVD QAVELTQEALGTVASQTRAVGERAAKLVGIELEAAAKAKFVAAWTLKAAAGGGSDAEIRTGPNAAY PVLYQVHKGDGGGSLDPGHGGSDQGASSRQGDKTLEKDIGGGSLETPNANGATVYWFHEQQEGL AGGGSTVMMKDKNHRQIVEQAIVDGLESYGGGSAKFVAAWTLKAAAGGGSEIPKINNEYLKEKRK KQRIQQRRVQRMIVGIHEYGAEALERAGDEVTIEKGLFNPIEVNVKEHHEYGAEALERAGVGAT FIPYDNVNNGQTSSASAKEVQSGTASEDKAKDDLQKALNKIKDEEHEYGAEALERAGAKFVAAWT LKAAAGGGS
3	V3	WP_014613729.1 (51–72, 124–148, 197–218, 265–288), and WP_130921585.1 (4–34, 100–119, 143–162) epitopes with HBHA-conserved adjuvant and PADRE sequence	EAAAKMAENSNIDDIKAPLLAALGAADLALATVNELITNLRERAEETRRSRVEESRARLTKLQEDLPE QLTELREKFTAEELRKAAEGYLEAATSELVERGEAALERLRSQQSFEEVSARAEGYVDQAVELTQE ALGTVASQVEGRAAKLVGIELEAAAKAKFVAAWTLKAAAGGGSDAEIRTGPNAAYPVLYQVHKGDG GGSLDPGHGGSDQGASSRQGDKTLEKDIGGGSLETPNANGATVYWFHEQQEGLAGGGSTVMMKD KNHRQIVEQAIVDGLESYGGGSAKFVAAWTLKAAAGGGSEIPKINNEYLKEKRKKQRIQQRRVQRM IVGIHEYGAEALERAGDEVTIEKGLFNPIEVNVKEHHEYGAEALERAGVGATFIPYDNVNNGQTS SASAKEVQSGTASEDKAKDDLQKALNKIKDEEHEYGAEALERAGAKFVAAWTLKAAAGGGS
4	V4	WP_014613729.1 (51–72, 124–148, 197–218, 265–288), and WP_130921585.1 (4–34, 100–119, 143–162) epitopes with Beta-defensin adjuvant and PADRE sequence	EAAAKGIINTLQKYYCRVRGGRCAVLSCLPKEEQIGKCSTRGRKCCRRKKEAAAKAKFVAAWTLK AAAGGGSDAEIRTGPNAAYPVLYQVHKGDGGGSLDPGHGGSDQGASSRQGDKTLEKDIGGGSL ETPNANGATVYWFHEQQEGLAGGGSTVMMKDKNHRQIVEQAIVDGLESYGGGSAKFVAAWT LKAAAGGGSEIPKINNEYLKEKRKKQRIQQRRVQRMIVGIHEYGAEALERAGDEVTIEKGLFNPIEV NVKEHHEYGAEALERAGVGATFIPYDNVNNGQTSSASAKEVQSGTASEDKAKDDLQKALNKIKD EEHEYGAEALERAGAKFVAAWTLKAAAGGGS
5	V5	WP_014613729.1 (76–121, 151–188, 227–251), and WP_130921585.1 (58–98, 143–162, 202–239) epitopes with L7/L12 Ribosomal protein adjuvant and PADRE sequence	EAAAKMSDLKNLAETLVNLTVKDVNELAAILKDEYGIEPAAAAVVMAGPGAEAAEEKTEFDVIL KSAGASKLAVVKLVKDLTGAGLKEAKDMVDGAPAAIKSGISKDEAEALKKQLEEAGAEVELKEA AAKAKFVAAWTLKAAAGGGSQIGKQGKWIEVRSANGKQKGWIAGWHTNLDIPADVNPHANP LRDKTIGGGSKTGLELKSLLEKKGAKVKMTRSTDEYVKLKDRNLKGDVGGGSKKAMLSNRGTRQ ENYQVLRQTEMPAGGGSAKFVAAWTLKAAAGGGSIKGNHYVSKQDILKELDIQNHPRIYAYSSDD AETRLKQNELHEYGAEALERAGDYKQEVPNEAPYIEGVKGAEHEYGAEALERAGDGIEVVGNT NTIAEKLKYYPSMSQALEKDETGKLKKSGHEYGAEALERAGAKFVAAWTLKAAAGGGS
6	V6	WP_014613729.1 (76–121, 151–188, 227–251), and WP_130921585.1 (58–98, 143–162, 202–239) epitopes with HBHA Adjuvant and PADRE sequence	EAAAKMAENPNIDDLPAPLLAALGAADLALATVNDLIANLRERAEETRAETRTRVEERRARLTKFQ EDLPEQFIELRDKFTTEELRKAAEGYLEAATNRYNELVERGEAALQRLRSQTAFEDASARAEGYV DQAVELTQEALGTVASQTRAVGERAAKLVGIELEAAAKAKFVAAWTLKAAAGGGSQIGKQGKW IEVRSANGKQKGWIAGWHTNLDIPADVNPHANPLRDKTIGGGSKTGLELKSLLEKKGAKVKMTR STDEYVKLKDRNLKGDVGGGSKKAMLSNRGTRQENYQVLRQTEMPAGGGSAKFVAAWTLK AAAGGGSIKGNHYVSKQDILKELDIQNHPRIYAYSSDDAETRLKQNELHEYGAEALERAGDYKQE VPNEAPYIEGVKGAEHEYGAEALERAGDGIEVVGNTNTIAEKLKYYPSMSQALEKDETGKL KKSGHEYGAEALERAGAKFVAAWTLKAAAGGGS
7	V7	130921585.1 (58–98, 143–162, 202–239) epitopes with HBHA-conserved adjuvant and PADRE sequence	EAAAKMAENSNIDDIKAPLLAALGAADLALATVNELITNLRERAEETRRSRVEESRARLTKLQEDL PEQLTELREKFTAEELRKAAEGYLEAATSELVERGEAALERLRSQQSFEEVSARAEGYVDQAVELTQE ALGTVASQVEGRAAKLVGIELEAAAKAKFVAAWTLKAAAGGGSQIGKQGKWIEVRSANGKQKGWI AGWHTNLDIPADVNPHANPLRDKTIGGGSKTGLELKSLLEKKGAKVKMTRSTDEYVKLKDRNLKG DVGGGSKKAMLSNRGTRQENYQVLRQTEMPAGGGSAKFVAAWTLKAAAGGGSIKGNHYVSK QDILKELDIQNHPRIYAYSSDDAETRLKQNELHEYGAEALERAGDYKQEVPNEAPYIEGVKGAEHEY GAEALERAGDGIEVVGNTNTIAEKLKYYPSMSQALEKDETGKLKKSGHEYGAEALERAGA KFVAAWTLKAAAGGGS
8	V8	WP_014613729.1 (76–121, 151–188, 227–251), and WP_130921585.1 (58–98, 143–162, 202–239) epitopes with Beta-defensin adjuvant and PADRE sequence	EAAAKGIINTLQKYYCRVRGGRCAVLSCLPKEEQIGKCSTRGRKCCRRKKEAAAKAKFVAAWTLK AAAGGGSQIGKQGKWIEVRSANGKQKGWIAGWHTNLDIPADVNPHANPLRDKTIGGGSKTGLEL KSLLEKKGAKVKMTRSTDEYVKLKDRNLKGDVGGGSKKAMLSNRGTRQENYQVLRQTEMPAG GGSAKFVAAWTLKAAAGGGSIKGNHYVSKQDILKELDIQNHPRIYAYSSDDAETRLKQNELHEYGA EALERAGDYKQEVPNEAPYIEGVKGAEHEYGAEALERAGDGIEVVGNTNTIAEKLKYYPSMS QALEKDETGKLKKSGHEYGAEALERAGAKFVAAWTLKAAAGGGS

### Analysis of chimeric vaccine constructs

The vaccine constructs (V1 to V8) generated from prioritized epitopes were assessed for physical and chemical characteristics. The aliphatic index represents the total space occupancy by aliphatic side chains, whereas the instability index (stability score <40.0) addresses the stability of vaccine constructs. GRAVY is another significant factor that is considered acceptable with negative scores only. It is calculated by estimating hydropathicity in amino acids by mean formula. For ease of *in vitro* vaccine development, final vaccine constructs should have a molecular weight of 41–59 kDa and amino acid composition range of 300–800 residues (Supplementary Table 6). Vaxijen v2.0 (score > 0.75) and ANTIGENpro (scoring > 0.90) estimated the antigenicity of the vaccine constructs. Furthermore, allergenicity was also determined using the AllergenFP server that utilizes potentially allergenic zones to identify allergenic proteins. Since the vaccine constructs will be expressed in a host system, such as *E. coli*, another criterion called solubility was examined. The solubility of vaccine constructs is important for vaccine production and it was determined by SOLpro server with a threshold of >0.5 (Supplementary Table 6). Following the analysis of all parameters, V2 was determined to be the best vaccine construct that fulfilled all the criteria, leading to future studies.

**FIGURE 3:**
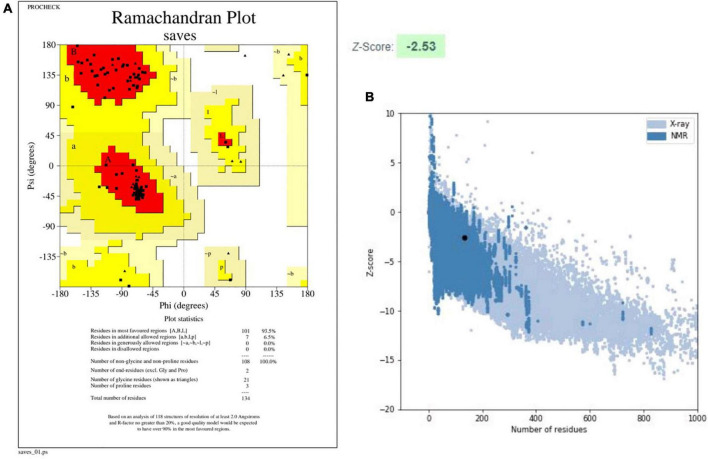
Validation results for the designed tertiary structure of vaccine construct (V2). **(A)** Ramachandran plot, **(B)** ProSA web graph.

The PSIPRED server was used to identify secondary structures in the finalized vaccine construct (Supplementary Figure 3), and the RaptorX property server was employed to estimate their attributes (Amineni et al., 2010). According to the RaptorX data, the vaccine construct’s predicted structure has 57% alpha-helix, 11% extended strand, and 30% coil structure. Additionally, solvent accessibility was envisaged by 50% of amino-acid residues being exposed, 23% moderately exposed, and 26% buried. The tertiary structure prediction was made by Phyre2, whereas the GalaxyRefine webserver was utilized to improve the accuracy of the modeled vaccine construct. The GalaxyRefine site developed five models after refining the first given vaccine structure. Depending on the parameters, the model 2 having GDT-HA (0.7705), RMSD (1.411), and MolProbity (2.261), was selected. The clashing value was obtained as 16.9, and the low rotamers value was estimated at 2.9, whereas Rama’s favored score was 97.0%. PROCHECK and ProSA web servers were used to further validate the model. Using the PROCHECK webserver, the improved structure was subjected to Ramachandran plot analysis. The plot revealed 93.5% amino acids in the preferable regions, 6.5% in permitted regions, and none in dis-allowed regions. The ProSA-webserver determined the Z score for the given vaccine construct to be 2.53 (Figure 3). The combined findings from the PROCHECK and ProSA-web servers verified the quality of the three-dimensional model of vaccine construct.

The disulfide bonds were introduced in predicted three-dimensional structure as they help to stabilize the 3D form in nature. The DbD2 server was employed for this server that discovered that 16 pairs of amino acids might form disulfide bonds in our vaccine construct (V2). However, after evaluating other factors like energy and chi3 value, only one pair was suggested for modification with cysteine. As a result, two mutations were produced at the LYS274–ARG277 residue pair. Energy and chi3 were selected with acceptable values of less than 2.2 and 87: +97, correspondingly.

### Molecular docking and MD simulation of vaccine construct

HLA molecules interact with linear T-cell epitopes, which trigger adaptive immunity against pathogens. The immunological response generated by epitopes might be physically limited, or epitopes could be detected by one person but not by another. As a result, a vaccine design should elicit an immune reaction in the presence of various HLA allele molecules. Different HLA allelic proportions of human populations may bind to the selected vaccine construct. We docked vaccine construct V2 with proteins from four distinct commonly occurring HLA alleles including 1A6A (HLA-DR B1*03:01), 3C5J (HLA-DR B3*02:02), 1AQD (HLA-DRB1*01:01), and 5NI9 (HLA-DR B1*04:01). The TLR4 complex interacts with the HBHA adjuvant, enhancing the immunological response; hence docking between the vaccine construct (V2) and TLR4 was also performed. The results indicated that the vaccine construct V2 and the TLR-4/MD2 complex had a positive interaction, with the highest docking score (Table 5).

**TABLE 5:** Docking analysis results of the best vaccine construct (V2) with different HLA alleles.

Vaccine construct	HLA alleles PDB ID’s	Score	Area	Hydrogen bond energy	Global energy	ACE
	1A6A	13918	1785.8	−1.57	−29.11	1.36
	1AQD	13116	1773.9	−2.28	−28.2	2.93
**V2**	5NI9	13976	1896.1	−2.47	−2.97	6.53
	3C5J	13530	1848.8	−2.59	−28.38	4.38
	**2Z63 (TLR4)**	**16102**	**2174.8**	−**1.67**	−**4.96**	**17.4**

**FIGURE 4:**
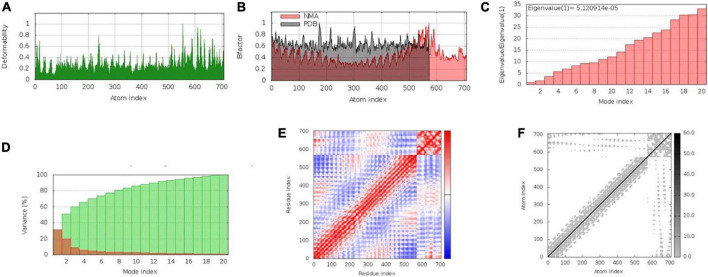
Molecular dynamics simulation of vaccine construct (V2)–TLR4 complex. The stability of the protein-protein complex was examined by **(A)** deformability, **(B)** B-factor values, **(C)** eigenvalue, **(D)** variance, **(E)** covariance of residue index, **(F)** elastic network analysis.

The iMODS server was used to execute normal mode analysis (NMA) for validation of binding affinity between the highest-scoring complex of vaccine construct and TLR4. The complex’s deformability is influenced by the perceived deformation of each residue, which is represented by hinges in the chains. The calculated eigenvalue, which indicates the complex movement rigidity, was 5.12e05. The eigenvalue and the variance associated with each standard mode were discovered to have an inverse relationship. The B-factor results were comparable to the RMS, and the covariance matrix depicted the connectivity between pairings of residues, with red, blue, and white indicating correlated, anti-correlated, and uncorrelated movements, respectively. The couples of atoms connected by springs were disclosed using an elastic network model, with stronger strings appearing as darker grays, proportional to the extent of stiffening between them (Figure 4).

### *In silico* cloning of vaccine construct

Codon adaptation is a method that optimizes codons for the bacterial genomes, potentially resulting in greater expression rates. This strategy was utilized to increase the vaccine construct synthesis rate in the *E. coli* K12 system since the codon usage of humans and targeted host differed. The JCat webserver was used to back-translate the sequence of the vaccine construct and modify the codons. The measured GC content was 52.04% upon analysis, which was acceptable as it was within the acceptable limit (30–70%). Likewise, the codon adaptation index (CAI) was determined to be 0.98, indicating a high level of vaccine construct expression. *In silico* cloning test by Snapgene revealed that the V2 vaccine construct could only clone into the pET28a (+) vector, ensuring heterologous cloning and production in the *E. coli* system (Supplementary Figure 4).

### Immune simulation of predicted vaccine construct

**FIGURE 5:**
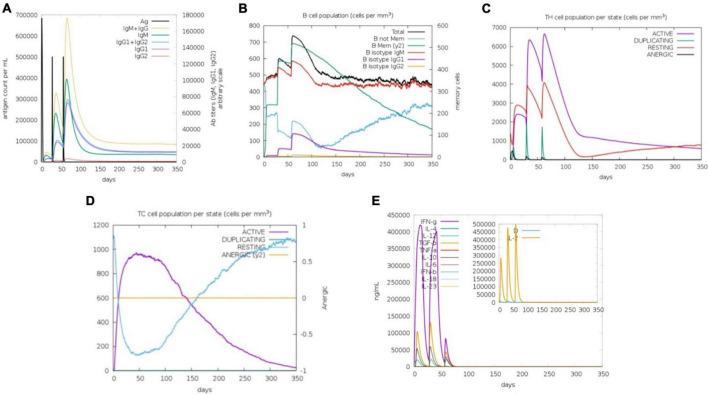
Immune simulation of vaccine construct (V2) in three injections by C-immsim server. **(A)** Primary response by antigen exposure, **(B)** B-cells immune reaction, **(C)** helper T-cells immune reaction, **(D)** cytotoxic T-cells immune reaction, **(E)** Simpson index D graph representing cytokines and Interleukin levels.

The C-ImmSim server simulated the body’s natural immunological reactions to the modeled vaccine construct. The first immune reaction is triggered by encounters with antigens, primarily generating IgM antibodies, while IgG is also released in small proportions. Antibodies are normally generated in low amounts, depending on the type of the antigenic pathogen. As a main immune reaction, the level of IgM began to rise by 1st administration of the vaccine dosage (antigens). Followed by two further antigen exposures with varying time intervals, a robust immune response develops that is characterized by greater levels of IgM and IgG. IgM, IgG1, and IgG1 + IgG2 levels, were observed to have increased significantly. This implied that the immunoglobulins had a higher binding affinity for the vaccine construct (antigens) and were more likely to establish immunological memory. As a response, additional antigenic doses resulted in higher pathogen elimination. In the case of CD8+ and CD+ T-cells, there was a strong reaction in different cells, which was accompanied by memory formation. The frequency of Helper T cells remained greater throughout the exposure. When cytokines levels elevated during the immune reaction, the Simpson index D graph assessed the amount of risk, which might result in human system complications (Figure 5).

## Discussion

The *S. pseudintermedius* species poses a significant risk of colonization in humans. This pathogen has genes with phenotypic characteristics that encourage staphylococcal skin and other infections. It also comprises gene sets that aid it in infecting and establishing itself amid natural microbiota by suppressing the growth of bacteria native to the area (Weese and Van Duijkeren, 2010). It is getting resistant to many antibiotics; thus, there is an emerging need for therapeutics that can affect multiple strains of this species. For this purpose, an extensive *in silico* analysis of the core-proteome obtained from 39 available complete genomes of this bacteria were performed. The aim was to determine novel drug target proteins as well as the vaccines against *S. pseudintermedius* species. The analysis was pursued by subtracting less significant proteins from various databases, including human proteome, essential genes, virulent factor proteins, antibiotic-resistant proteins, human interacting proteins, and the gut microbiome. Resultant proteins were further checked for their physicochemical characteristics to reduce the number for laboratory production of potential drug targets. The three-dimensional structures were not available for the predicted drug targets; thus, homology modeling was performed to analyze the drug pockets and shortlist significant drug targets based on drug binding screening. Among final potential drug targets, SUF system NifU family Fe-S cluster assembly protein (WP_014614444.1), MULTISPECIES: Ribonuclease-P protein component (WP_015728500.1), YvcK family protein (WP_198461100.1), DUF4479 domain-containing protein (WP_014613626.1), and UDP-N-acetylmuramoyl-L-alanyl-D-glutamate-L-lysine ligase (WP_063284624.1) were obtained with good druggability scores.

The NifU (nitrogen fixation) family Fe-S cluster assembly protein (WP 014614444.1) from the SUF (sulfur assimilation) system is involved in the binding of the Fe-S cofactors that are the key co-factors for a range of proteins participating in electron transfer, redox and non-redox catalysis, gene regulation, and as oxygen and iron sensors. Since this numerous FeS cluster prosthetic groups are required for significant roles, their interaction enzymes are engaged in a variety of key biological functions and are considered as vital drug targets (Ouzounis et al., 1994).

The ribonuclease-P protein component (WP 015728500.1) catalyzes removal of the 5′-leader sequence from pre-tRNA to form the mature 5′-terminus by combining a catalytic RNA component (M1 or rnpB) with a protein subunit. Other RNA substrates, such as 4.5S RNA, can also be cleaved. RNase P is made up of two parts in bacteria: a large RNA (about 400 base pairs) encoded by rnpB and a small protein (119 to 133 amino acids) encoded by rnpA. *In vivo*, the protein component serves as an auxiliary but critical component by attaching to the 5′-leader sequence and widening the ribozyme’s substrate selectivity (Brown and Pace, 1992).

The YvcK family protein has a strong similarity to the Gluconeogenesis factor, which comprises the 2-phospho-L-lactate transferase CofD. CofD catalyzes the transfer of the 2-phospholactate moiety from lactyl (2) diphospho-(5′) guanosine (LPPG) to 7,8-didemethyl-8-hydroxy-5-deazariboflavin (FO) (Forouhar et al., 2008).

The domain-containing protein DUF4479 (WP 014613626.1) is linked to the recognition of the L shape of tRNA. The TRBD domain has been shown to have a general tRNA-binding capacity, and it has been proposed that the TRBD domain could be a non-specific tRNA binder acting in cis or trans to improve the catalytic efficiency or substrate specificity of aminoacyl-tRNA synthetases (Simos et al., 1996).

The MurM and MurC domains of the UDP-N-acetylmuramoyl-L-alanyl-D-glutamate-L-lysine ligase (WP 063284624.1) catalyze sequential stages in the peptidoglycan synthesis. Peptidoglycan is made up of two sugar derivatives, one of which is N-acetylmuramic acid, attached to a short pentapeptide. L-alanine, D-glutamic acid, Meso-diaminopimelic acid, and D-alanyl alanine make up the pentapeptide. By gradually adding these amino acids to UDP-N-acetylmuramic acid, the peptide moiety is created. L-alanine is transferred by MurC, whereas the other amino acid is transferred by MurM (Bertrand et al., 1997). All predicted proteins were found to be involved in the significant functions of pathogen life cycle and thus could be used as potential drug targets against *S. pseudintermedius* species.

Furthermore, extracellular proteins were subjected to potential vaccine target analysis. Various parameters were employed for the selection of the best potential vaccine candidates including Protparam analysis, antigenicity determination and transmembrane helices determination. After analysis, two proteins were preferred and proceeded for chimeric vaccine designing. It was done by predicting top affinity T and B-cell epitopes. They were screened for antigenic, non-toxic, conserved and non-allergenic attributes before final selection. The best final epitopes were combined with linkers and adjuvant sequence was added to boost the immune response. These constructed vaccine designs were evaluated by various computational methods, i.e., the physicochemical, structural, and immunological features of the vaccine constructs were investigated. Out of all vaccine constructs, one was finally selected after the analysis. It was discovered to be stable, hydrophilic, soluble, antigenic, and non-allergenic. Its structural validation using Ramachandran plots and ProSA demonstrated that a high-quality 3D structure had been built. The suggested vaccine’s molecular docking with TLR4 yielded a low energy and high docking score, indicating strong binding affinity. Docked protein complex exhibit minimal deformability, as indicated by a molecular dynamics simulation, demonstrating the validity of our predicted vaccine. This final vaccine construct can be forwarded to laboratory for further validation and it may be helpful in future vaccine development to combat *S. pseudintermedius* infections.

## Conclusion

Prior to biological tests, relying on contemporary *in silico* methodologies for drug and vaccine research is a successful strategy that may direct studies with a high likelihood of identifying effective medications and vaccines with fewer trials. In this study, subtractive proteomics and reverse vaccinology approach was applied to the core proteome of 39 *S. pseudintermedius* complete genomes, to prioritize potential novel druggable proteins as well as design a chimeric vaccine construct for humans. Application of different rigorous analysis resulted in the identification of two pathway dependent and 32 pathway independent proteins as potential drug targets. All of the identified drug target proteins were found important for bacterial growth, survival, and virulence, and could be used as therapeutic targets in the future.

After screening, two extracellular proteins (WP 014613729.1 and WP 130921585.1) were identified as best potential vaccine target candidates. Employing different tools, B-cell and T-cell epitopes of these two proteins were determined. The shortlisted epitopes were combined and eight vaccine constructs were designed and subjected to physical and chemical analysis. Finally, a vaccine construct namely V2 was determined as the best candidate, for which secondary and tertiary structure analysis were performed. The elicitation of the human immune response by this construct was confirmed by docking and MD simulation analysis with four distinct HLA alleles. Lastly, cloning and expression of the potential vaccine construct was predicted in *E. coli* system while immune simulation analysis confirmed that immunoglobulins have a higher binding affinity for this construct. Further experimental testing and validation of these multi-epitope constructs might be beneficial to combat the *S. pseudintermedius* infections.

## Data availability statement

The original contributions presented in this study are included in the article/Supplementary material, further inquiries can be directed to the corresponding authors.

## Author contributions

MS and SO contributed to resources, conceptualization, supervision and editing of the manuscript. SJ, AA, and MJ performed the experiments. UN, GM, HN and SO wrote the manuscript. SA and NU proofread the final version. All authors contributed to the article and approved the submitted version.

## Supplementary material

Supplementary Figure 1 Heat map illustration showing cluster analysis of HLA alleles for both MHC molecules. The MHC-I cluster is shown in **A** while the MHC-II molecules cluster is being represented in **B**. Epitopes are grouped according to their association with HLA-alleles, especially red suggesting a significant interaction. The weaker interaction is indicated by the yellow zone.

Supplementary Figure 2 Predicted Linear B-cell epitopes analysis including Emini Surface Accessibility Prediction, Karplus and Schulz Flexibility Prediction, Kolaskar and Tongaonkar Antigenicity, Parker Hydrophilicity Prediction, Bepipred Linear Epitope, and Chou and Fasman Beta-Turn Prediction. **A-1–A-6** represents the analysis of WP 014613729.1 while **B-1–B-6** shows analysis graphs of WP 130921585.1 protein.

Supplementary Figure 3 Secondary structures (Alpha helixes, extended strands, and Beta sheets) prediction using PSIPRED v4.0 server.

Supplementary Figure 4 *In silico* restriction cloning of final vaccine construct (V2) into the *E. coli* pET28a (+) expression vector where red color shows the cloned vaccine construct.
