# Supplementary material for: Multi-epitope chimeric vaccine designing and novel drug targets prioritization against multi-drug resistant Staphylococcus pseudintermedius
Source: Front Microbiol. 2022 Aug 4;13:971263. doi: 10.3389/fmicb.2022.971263 (PMC9386485; doi:10.3389/fmicb.2022.971263)
Supplement: Supplementary file 3 [file Table_3.docx]

**Table S3.** MHC-I interacting T-cell epitopes for the two potential vaccine candidate proteins.

| **Protein IDs** | **Sr No.** | **Epitopes** | **Start** | **End** | **MHC-I alleles** | **Predicted score** | **Percentile rank** | **Antigenicity** | **Toxicity** | **Immunogenicity** | **Conservancy analysis** |
| --- | --- | --- | --- | --- | --- | --- | --- | --- | --- | --- | --- |
| **WP 014613729.1** | 1 | AAYPVLYQV | 60 | 68 | HLA-A*02:06 | 0.980247 | 0.01 | Antigen | Non-Toxin | -0.0534 | 88.89% |
|  | **2** | **KRIHPQRFY** | **11** | **19** | **HLA-C*07:01** | **0.595798** | **0.01** | **Antigen** | **Non-Toxin** | **0.06799** | **100.00%** |
|  | 3 | QTLNASIQK | 219 | 227 | HLA-A*11:01 | 0.953165 | 0.01 | Antigen | Non-Toxin | -0.0831 | 100.00% |
|  | 4 | RQTEMPAVL | 245 | 253 | HLA-B*48:01 | 0.914262 | 0.01 | Antigen | Non-Toxin | -0.01095 | 100.00% |
|  | 5 | AIVDGLESY | 280 | 288 | HLA-B*15:01 | 0.914158 | 0.02 | Non-Antigen | Non-Toxin | 0.04612 | 100.00% |
|  | 6 | KQKGWIAGW | 92 | 100 | HLA-A*32:01 | 0.766502 | 0.02 | Non-Antigen | Non-Toxin | 0.3579 | 100.00% |
|  | **7** | **NANGATVYW** | **201** | **209** | **HLA-B*53:01** | **0.92251** | **0.02** | **Antigen** | **Non-Toxin** | **0.13932** | **100.00%** |
|  | 8 | YVKLKDRNL | 176 | 184 | HLA-B*08:01 | 0.88272 | 0.02 | Antigen | Non-Toxin | -0.23038 | 100.00% |
|  | **9** | **KTLEKDITL** | **142** | **150** | **HLA-E*01:03** | **0.256744** | **0.03** | **Antigen** | **Non-Toxin** | **0.04303** | **100.00%** |
|  | **10** | **IALVGILVV** | **25** | **33** | **HLA-B*51:01** | **0.594475** | **0.13** | **Antigen** | **Non-Toxin** | **0.21098** | **100.00%** |
| **WP 130921585.1** | 1 | EIPKINNEY | 4 | 12 | HLA-A*26:01 | 0.950215 | 0.01 | Antigen | Non-Toxin | -0.04405 | 100.00% |
|  | **2** | **SSDDAETRL** | **85** | **93** | **HLA-C*05:01** | **0.987192** | **0.01** | **Antigen** | **Non-Toxin** | **0.22487** | **100.00%** |
|  | 3 | TIIAITTEK | 120 | 128 | HLA-A*11:01 | 0.921783 | 0.01 | Non-Antigen | Non-Toxin | 0.33997 | 100.00% |
|  | 4 | YYPSMSQAL | 219 | 227 | HLA-C*14:02 | 0.984337 | 0.01 | Antigen | Non-Toxin | -0.5717 | 100.00% |
|  | 5 | FMRDGIEVV | 199 | 207 | HLA-A*02:03 | 0.947706 | 0.02 | Non-Antigen | Non-Toxin | 0.30602 | 100.00% |
|  | 6 | SADIKGNHY | 55 | 63 | HLA-A*01:01 | 0.944314 | 0.02 | Antigen | Non-Toxin | -0.02354 | 100.00% |
|  | 7 | VLKDYKQEV | 140 | 148 | HLA-A*02:03 | 0.927406 | 0.03 | Non-Antigen | Non-Toxin | -0.29354 | 100.00% |
|  | **8** | **AQISEIVSA** | **179** | **187** | **HLA-A*02:06** | **0.904152** | **0.04** | **Antigen** | **Non-Toxin** | **0.03769** | **100.00%** |
|  | 9 | AKDDLQKAL | 278 | 286 | HLA-B*48:01 | 0.302223 | 0.1 | Non-Antigen | Non-Toxin | -0.24946 | 100.00% |
|  | 10 | RVQRMIVGI | 26 | 34 | HLA-A*32:01 | 0.456297 | 0.13 | Non-Antigen | Non-Toxin | 0.0234 | 100.00% |

**Table S3.** MHC-II interacting T-cell epitopes for the two for the two potential vaccine candidate proteins.

| **Protein IDs** | **S/No** | **Epitopes** | **Start** | **End** | **MHC-II alleles** | **Antigenicity** | **Toxicity** | **IFN Inducers** | **IL4 Inducer** | **IL10 Inducer** | **Conservancy analysis** |
| --- | --- | --- | --- | --- | --- | --- | --- | --- | --- | --- | --- |
| **WP 014613729.1** | 1 | ALVGILVVSYFVLRH | 26 | 40 | HLA-DPA1*01/DPB1*04:01 | Antigen | No | Positive | No | No | 100.00% |
|  | 2 | ELGYISNPTDTVMMK | 255 | 269 | HLA-DRB3*02:02 | Non | No | Positive | Yes | No | 93.33% |
|  | 3 | ETPNANGATVYWFHE | 198 | 212 | HLA-DQA1*01:02/DQB1*06:02 | Antigen | No | Negative | Yes | No | 93.33% |
|  | 4 | KKAMLSNRGTRQENY | 227 | 241 | HLA-DRB3*02:02 | Antigen | No | Negative | No | Yes | 100.00% |
|  | 5 | IEVRSANGKQKGWIA | 84 | 98 | HLA-DRB5*01:01 | Antigen | No | Positive | Yes | No | 100.00% |
|  | 6 | KDRNLKGDVFISIHN | 180 | 194 | HLA-DPA1*02:01/DPB1*01:01 | Antigen | No | Negative | No | No | 100.00% |
|  | 7 | SDDDHINLIEDAEIR | 41 | 55 | HLA-DQA1*03:01/DQB1*03:02 | Antigen | No | Positive | Yes | No | 100.00% |
|  | 8 | GWIAGWHTNLDIPAD | 95 | 109 | HLA-DQA1*01:01/DQB1*05:01 | Antigen | No | Positive | No | No | 100.00% |
|  | 9 | DKNHRQIVEQAIVDG | 270 | 284 | HLA-DRB4*01:01 | Antigen | No | Negative | No | No | 100.00% |
|  | 10 | DKTLEKDITLKTGLE | 141 | 155 | HLA-DRB1*03:01 | Antigen | No | No Data | Yes | No | 100.00% |
| **WP 130921585.1** | 1 | VGILVVIVLLILVYM | 32 | 46 | HLA-DPA1*03:01/DPB1*04:02 | Antigen | No | Positive | No | Yes | 100.00% |
|  | 2 | AEKLKYYPSMSQALE | 214 | 228 | HLA-DRB1*09:01 | Antigen | No | Negative | Yes | No | 100.00% |
|  | 3 | HTIIAITTEKSRVVP | 119 | 133 | HLA-DRB1*08:02 | Non | No | No Data | Yes | No | 100.00% |
|  | 4 | FTPISHIKSADIKGN | 47 | 61 | HLA-DRB1*07:01 | Antigen | No | Positive | Yes | No | 100.00% |
|  | 5 | KRKKQRIQQRRVQRM | 16 | 30 | HLA-DRB4*01:01 | Antigen | No | No Data | Yes | No | 100.00% |
|  | 6 | PHLIKLFMRDGIEVV | 193 | 207 | HLA-DRB3*01:01 | Non | No | No Data | Yes | No | 100.00% |
|  | 7 | ENGKVLKDYKQEVPN | 136 | 150 | HLA-DRB1*03:01 | Antigen | No | Positive | No | No | 100.00% |
|  | 8 | QDILKELDIQNHPRI | 67 | 81 | HLA-DRB4*01:01 | Antigen | No | Positive | Yes | Yes | 100.00% |
|  | 9 | GKLKKSGFIDLSVGA | 233 | 247 | HLA-DQA1*01:01/DQB1*05:01 | Antigen | No | Negative | Yes | Yes | 100.00% |
|  | 10 | NNEYLKEKRKKQRIQ | 9 | 23 | HLA-DRB1*11:01 | Antigen | No | Positive | Yes | Yes | 100.00% |
