# Supplementary figures and images for: Multi-epitope chimeric vaccine designing and novel drug targets prioritization against multi-drug resistant Staphylococcus pseudintermedius
Source: Front Microbiol. 2022 Aug 4;13:971263. doi: 10.3389/fmicb.2022.971263 (PMC9386485; doi:10.3389/fmicb.2022.971263)

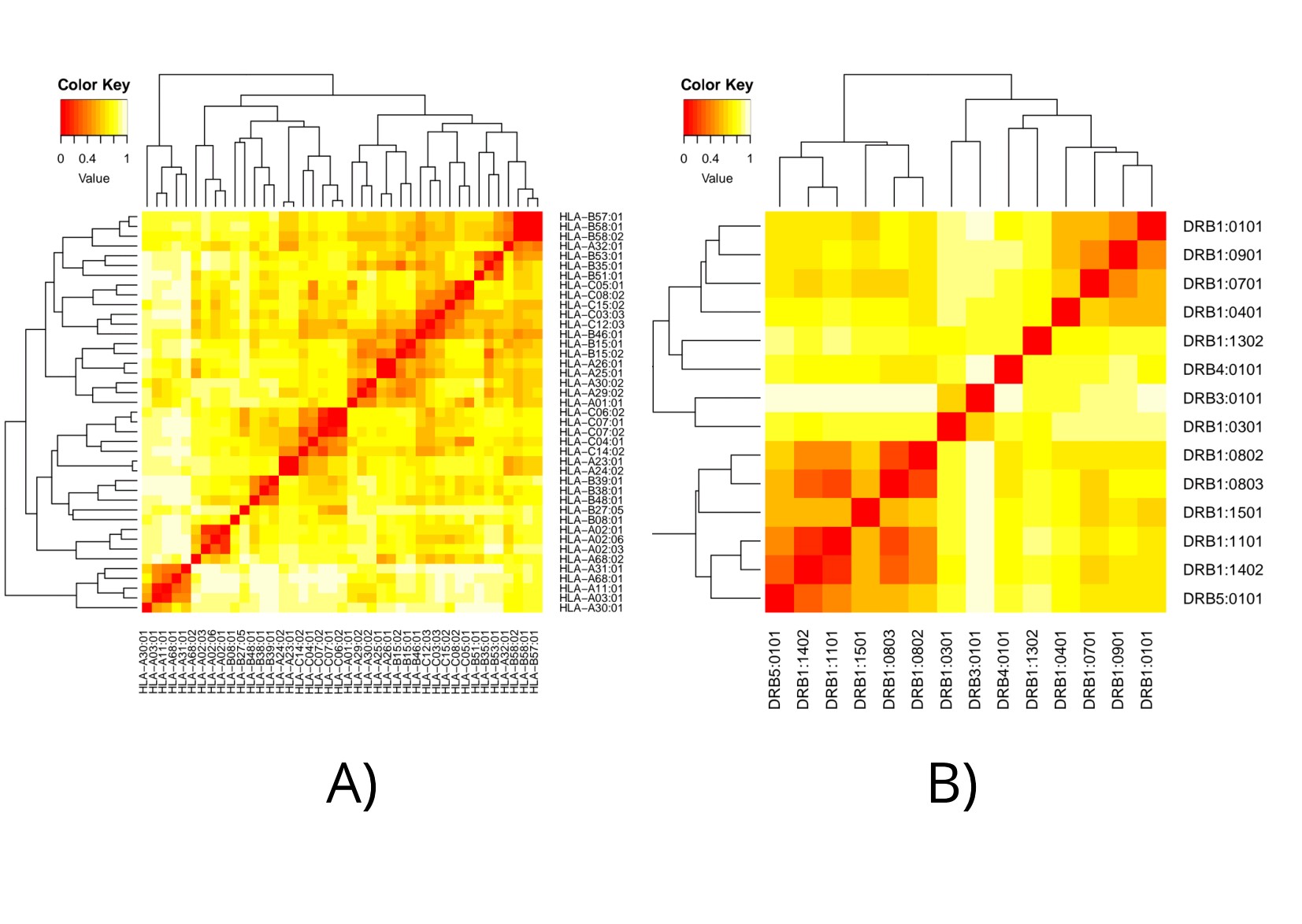

Supplement: Supplementary Figure 1 — Heat map illustration showing cluster analysis of HLA alleles for both MHC molecules. The MHC-I cluster is shown in A while the MHC-II molecules cluster is being represented in B. Epitopes are grouped according to their association with HLA-alleles, especially red suggesting a significant interaction. The weaker interaction is indicated by the yellow zone. [file Image_1.JPEG]

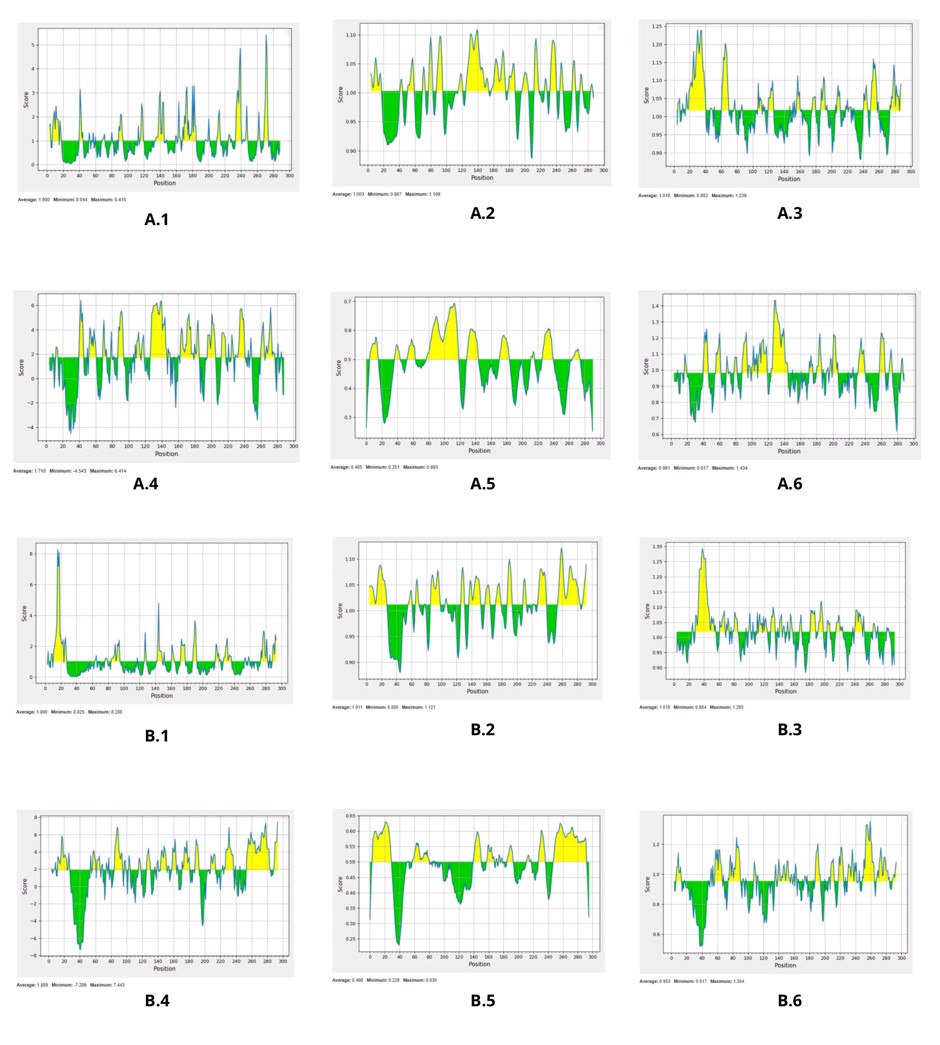

Supplement: Supplementary Figure 2 — Predicted Linear B-cell epitopes analysis including Emini Surface Accessibility Prediction, Karplus and Schulz Flexibility Prediction, Kolaskar and Tongaonkar Antigenicity, Parker Hydrophilicity Prediction, Bepipred Linear Epitope, and Chou and Fasman Beta-Turn Prediction. A-1–A-6 represents the analysis of WP 014613729.1 while B-1–B-6 shows analysis graphs of WP 130921585.1 protein. [file Image_2.JPEG]

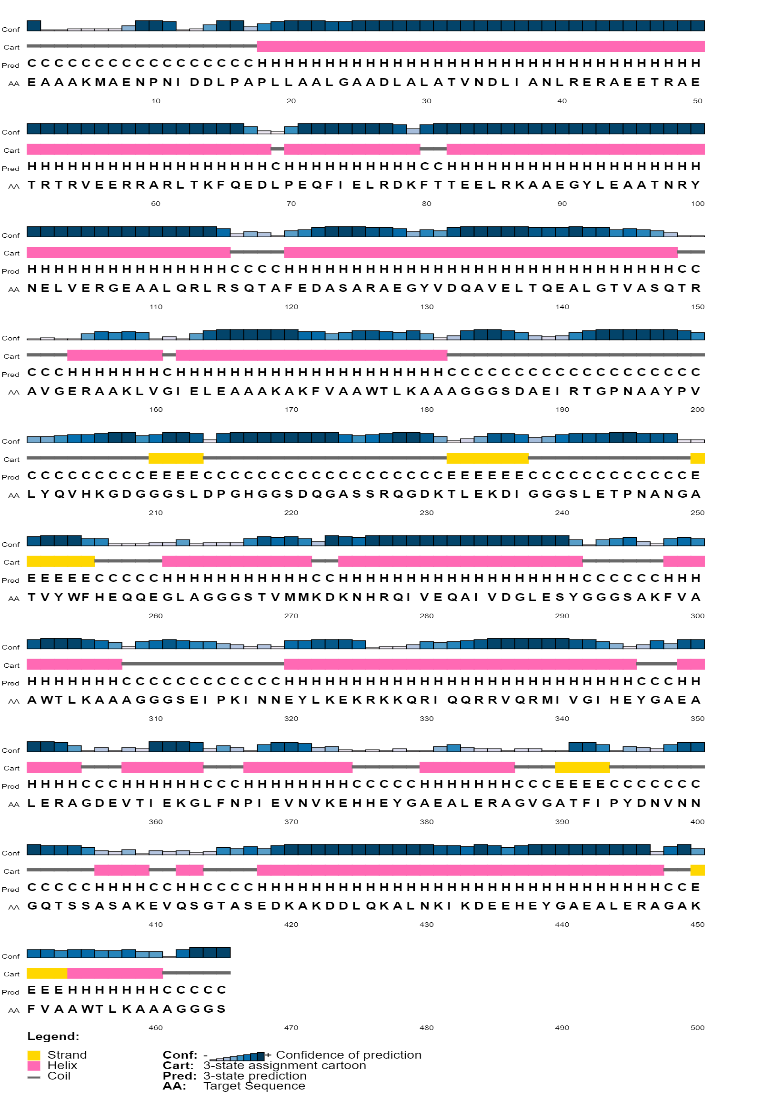

Supplement: Supplementary Figure 3 — Secondary structures (Alpha helixes, extended strands, and Beta sheets) prediction using PSIPRED v4.0 server. [file Image_3.PNG]

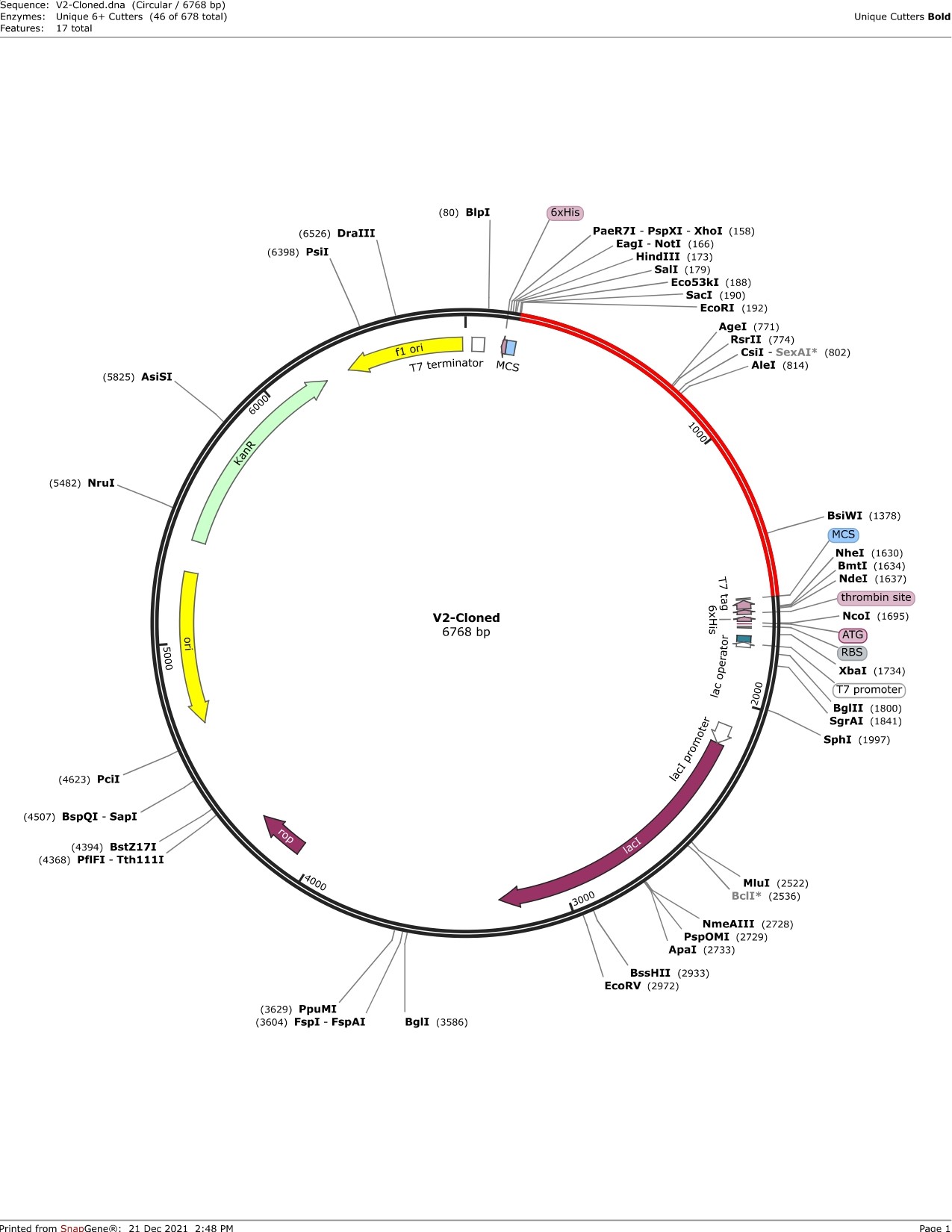

Supplement: Supplementary Figure 4 — In silico restriction cloning of final vaccine construct (V2) into the E. coli pET28a (+) expression vector where red color shows the cloned vaccine construct. [file Image_4.JPEG]
